# Sensors, Circuits, and Systems for Biomedical Applications

**DOI:** 10.3390/s23063295

**Published:** 2023-03-21

**Authors:** Jungsuk Kim

**Affiliations:** Department of Biomedical Engineering, Gachon University, Incheon 21936, Republic of Korea; jungsuk@gachon.ac.kr

Sensor technologies (including electrodes) have been widely utilized in many applications, especially in fields such as smart factories, automation, clinics, laboratories, and more. Today, advances in microelectromechanical system (MEMS) and complementary metal oxide semiconductor (CMOS) technologies enable sensors to be miniaturized, and as a result, we can increase the number of sensors available in a limited space Accordingly, it is essential to realize low-power, low-noise, multi-channel electronic devices, which have a high-density sensing array, in order to diagnose diseases, restore neurological and muscular disorders, analyze DNA molecules.

This Special Issue presents several examples of articles that focus on biomedical applications, for example: flexible/wearable/implantable sensors, circuits, and systems; integrated circuits and systems for biomedical applications; high-precision circuits and systems for DNA/RNA molecule analysis; biomedical sensors for disease diagnosis; electrodes and stimulators for biomedical applications; low-power, low-noise, and multi-channel circuits for neural interfaces; and wireless telemetry circuits and systems for biomedical applications.

In recent years, numerous studies have demonstrated the beneficial effects of administering appropriate electrical stimulation to humans, such as for treating diseases, reducing pain, and replacing the function of existing nerves [1,2,3,4]. To provide a glimpse into the ongoing research on electrical stimulation, we introduce several examples of spinal cord stimulation and retina prosthesis research. Yun S. et al. developed a fully implantable, miniaturized spinal cord stimulator based on liquid crystal polymers (LCPs) for pain control, which addresses the bulky size and heavy weight limitations of the existing implantable pulse generators (IPGs) used for spinal cord stimulation [1]. Experimental testing conducted by implanting the developed system into mice has revealed that it outperforms previous systems in terms of pain suppression efficacy.

Zawawi R.B.A. et al. proposed the addition of back telemetry to a retina prosthesis system which wirelessly transmits power, allowing the external transmitter to confirm whether the transmitted power is being received successfully by the system [2]. Moreover, in the event of abnormal reception, the receiver is designed to stop functioning until successful reception is achieved. On the other hand, Abbasi W. et al. presented an economical and configurable wireless communication system that uses field-programmable gated arrays (FPGAs) to address the design challenges associated with retinal implantation [3]. This system utilizes hexagonal biphasic stimulation pulses that are generated by a digital controller, which can be fully controlled using an external transmitter. Both studies address potential safety concerns and ensure the reliable operation of the retina prosthesis system. Kang H. et al. set out to overcome the technical challenges related to ambient light and current dispersion in high-density retinal implants and proposed a novel image-processing unit in the form of an ALR circuit for sub-retinal implants, which can handle ambient light, while considering power and area constraints [4].

A number of investigations are currently underway to overcome the issues associated with measuring signals from the human body and existing signal measurement methods. In this Special Issue, four articles focus on this issue [5,6,7,8]. Ben Atitallah B. et al. present a systematic comparison of methods for bioimpedance spectroscopy (BIS) measurement, including the use of a gain phase detector (GPD), IQ demodulation, and fast Fourier transform (FFT), with a focus on magnitude and phase measurement accuracy, signal processing, hardware and software complexity, and power consumption [5]. The results demonstrate that the FFT-based method achieves high accuracy for amplitude and phase measurement, with low hardware complexity and power consumption but at the cost of higher software complexity. Kim Y.J. et al. proposed an enzyme-free electrochemical wireless sensor for continuous glucose monitoring, which is based on a microneedle array with a 3 × 5 stainless steel electrode configuration that is easily miniaturized for painless monitoring [6]. Rajendran D. et al. developed flexible and low-cost in-shoe sensors based on multi-walled carbon nanotubes (MWCNT)/ polydimethylsiloxane (PDMS) for accurate foot pressure monitoring, which possess excellent electrical and mechanical properties and demonstrate a stable response under constant pressure loadings for over 5000 cycles [7]. Bednar T. et al. explored the use of capacitive bio-signal measurement for long-term and wearable monitoring but highlighted its susceptibility to environmental noise, particularly power-line noise. Their paper describes the origin of noise and proposes two electric models for its reduction: a passive capacitive grounding electrode and an active capacitive driven right leg (DRL) electrode [8].

This Special Issue also includes engineering research for various disciplines such as neuroscience, genetics, and developmental biology [9,10]. Hong J. et al. proposed a low-area multi-channel controlled dielectric breakdown (CDB) system and the system is proposed for the simultaneous production of several nanopore sensors to address the challenges associated with pore-to-pore diameter variation in solid-state nanopores [9]. Lee Y. et al. developed a modular microfluidic chip that allows for uniform and continuous drug infusion across multiple channels during EEG recordings, enabling easy scalability for investigations with a larger number of animals [10]. Such biomedical application tools that offer benefits such as having a compact size and low fabrication costs are highly desirable for practical applications in the field.

In conclusion, this Special Issue highlights the latest research being carried out on sensors, circuits, and systems for biomedical applications. The field of biomedical applications is advancing at a rapid pace, and its impact is palpable in our daily lives. Through this Special Issue, we intend to provide guidance for the development of sensors, circuits, and systems for biomedical applications, with the ultimate goal of facilitating progress in this area.

## Data Availability

Not applicable.
